# Variation in Event-Related Potentials by State Transitions

**DOI:** 10.3389/fnhum.2017.00075

**Published:** 2017-02-27

**Authors:** Hiroshi Higashi, Tetsuto Minami, Shigeki Nakauchi

**Affiliations:** ^1^Department of Computer Science and Engineering, Toyohashi University of TechnologyAichi, Japan; ^2^Electronics-Inspired Interdisciplinary Research Institute, Toyohashi University of TechnologyAichi, Japan

**Keywords:** Event-Related Potentials (ERPs), Electroencephalography (EEG), predictive surprise, model-based analysis, single-trial analysis

## Abstract

The probability of an event's occurrence affects event-related potentials (ERPs) on electroencephalograms. The relation between probability and potentials has been discussed by using a quantity called surprise that represents the self-information that humans receive from the event. Previous studies have estimated surprise based on the probability distribution in a stationary state. Our hypothesis is that state transitions also play an important role in the estimation of surprise. In this study, we compare the effects of surprise on the ERPs based on two models that generate an event sequence: a model of a stationary state and a model with state transitions. To compare these effects, we generate the event sequences with Markov chains to avoid a situation that the state transition probability converges with the stationary probability by the accumulation of the event observations. Our trial-by-trial model-based analysis showed that the stationary probability better explains the P3b component and the state transition probability better explains the P3a component. The effect on P3a suggests that the internal model, which is constantly and automatically generated by the human brain to estimate the probability distribution of the events, approximates the model with state transitions because Bayesian surprise, which represents the degree of updating of the internal model, is highly reflected in P3a. The global effect reflected in P3b, however, may not be related to the internal model because P3b depends on the stationary probability distribution. The results suggest that an internal model can represent state transitions and the global effect is generated by a different mechanism than the one for forming the internal model.

## 1. Introduction

Humans make predictions by using prior information (Doya et al., 2007; Friston, 2008), and the prior information is derived from what humans have experienced. The prediction of an event's occurrence is equivalent to the estimation of the generative model for the event (Robert, 2007). However, the manner in which one utilizes that experience in estimating the generative model remains unclear.

One approach for revealing how experience affects human prediction is the observation of event-related potentials (ERPs). ERPs are the measured brain responses for a specific internal or external event (Clark, 2013). Some ERP components observed on electroencephalograms (EEGs) are affected by the probability of the occurrence of the event (Sutton et al., 1965; Squires et al., 1977; Picton, 1992). In particular, a slow variation observed about 300 ms after the event on the EEG potentials is called P300, and its peak amplitude depends on the probability of the event's occurrence (Duncan-Johnson and Donchin, 1977). Such ERP components are considered to reflect the process for predicting events and are widely used as a medium for analyzing human cognition (Horovitz et al., 2002; Sanmiguel et al., 2013).

The relation between these ERP components and probability has been discussed by using a quantity called *the degree of surprise* or simply *surprise* (Ostwald et al., 2012). One concept of surprise is called *predictive surprise* that represents the subjective self-information, information content, or surprisal (Shannon, 1948) that an observer receives from an observed event (Donchin and Coles, 1988). Recently, Mars et al. (2008) and Kolossa et al. (2012) addressed the question of which factors in a preceding stimulus sequence affect predictive surprise to a present stimulus by investigating the relation between the stimulus sequence and P300 properties. To identify these factors, Mars et al. (2008) and Kolossa et al. (2012) used regression models in which the input was a stimulus history and the output was the P300 amplitude. Their approach with these regression models is called *the model-based approach*. Assuming that the observed brain activities reflect the prediction process, the model-based approach can confirm which factors human prediction depends on by finding a model that accurately estimates the amplitude of P300. Their results suggest that surprise estimated by the integration of three factors (long-term history, short-term history, and alternating expectations of the stimulus sequence) adequately predicts the P300 amplitude (Squires et al., 1976; Kolossa et al., 2012).

The other concept of surprise, proposed by Baldi and Itti (2010), is called *Bayesian surprise* that represents the degree of updating in the beliefs of an observer who experiences a new event. Recent studies (Kolossa et al., 2015; Seer et al., 2016) showed that predictive and Bayesian surprises affect different subcomponents of P300 called P3a and P3b (Polich, 2007). Predictive surprise better explains P3b, which has a long latency among the subcomponents. However, Bayesian surprise better explains P3a, which has a short latency. The results suggest that the subcomponents reflect distinct neural mechanisms for prediction.

To reveal the relation between ERPs and surprise, theoretical frameworks, such as the context-updating model (Donchin, 1981; Donchin and Coles, 1988; Polich, 2007), predictive coding (Friston, 2002; Spratling, 2010), and Bayesian brain hypothesis (Hampton et al., 2006; Kopp, 2006; Ostwald et al., 2012; Lieder et al., 2013), have been convincingly established. The frameworks explain human behavior or brain responses by positing the existence of an internal model that humans constantly and automatically generate about the external world (Donchin, 1981). Different processes of the response of the internal model to an external event lead to different brain activities, such as the P3a and P3b variations (Kolossa et al., 2015).

What state transition the internal model builds is discussed in this study. Previous studies, such as Mars et al. (2008) and Kolossa et al. (2012), assumed that the internal model is without state transitions. Accordingly, surprises were estimated based on a generative model in a stationary state (a stationary-state model). In contrast, the purpose of our study is to find evidence of a brain mechanism that codes state transitions. If the brain can generate an internal model with state transitions (the state transition model), then humans would not acquire a probability distribution of events but would acquire a model that describes how different states or situations of the world are connected to each other (Gläscher et al., 2010).

The possibility that the state transition models explain some effects in ERP components motivated this study. These properties of an event sequence, such as stationary-state models, alternation, and repetition (Matt et al., 1992; Rac-Lubashevsky and Kessler, 2016) that explain the variation in some ERP components can be generalized with a state transition model. Moreover, Gläscher et al. (2010) suggested that probability distributions with state transitions are coded in the brain during the performance of reinforcement learning tasks (Saito et al., 2015). Therefore, we hypothesized that, for prediction, a mechanism for coding state transitions exist; that is, surprise is modeled not only with the probability distribution of the stationary state but also with the probability distribution with state transitions.

In the present study, we investigated the relation between predictive surprise in a generative model that has state transitions and electrophysiological signals via a model-based analysis. To distinguish predictive surprises in the different state models, predictive surprise with state transitions is called *predictive transition surprise*, and predictive surprise in a stationary state is called *predictive stationary surprise*. Although the EEG signals were recorded with a two-choice response time task, the same one used by Mars et al. (2008) and Kolossa et al. (2012), the generative models for the event sequences were different. In previous studies, predictive transition surprise converged with predictive stationary surprise as the number of trials increased; therefore, that type of setting cannot isolate the effects of predictive transition surprise. To avoid this situation, we used state transition models for the generative models; we controlled generation of the event sequence with a simple Markov chain (Norris, 1998). In the model-based analysis, we used predictive stationary or transition surprise of the Markov chain as the explanatory variable. As the response variable, we used the EEG potentials, which were observed in various electrodes and latencies. This analysis enabled us to visualize the effects of these surprises on various ERP components, such as P3a and P3b. The results show different brain activities that seem to be associated with the stationary-state model and the state transition model.

## 2. Materials and methods

### 2.1. Measurement

#### 2.1.1. Participants

Twelve individuals (10 male and 2 female) participated in the experiment. Their ages ranged from 21 to 27 years (*M* = 23.6; *SD* = 1.7). The participants had normal or corrected-to-normal visual acuity. All participants provided written informed consent. The experimental protocols were approved by the Committee for Human Research at the Toyohashi University of Technology, Aichi, Japan, and the experiment was conducted in accordance with the committee's approved guidelines.

#### 2.1.2. Experimental design

**Figure 1:**
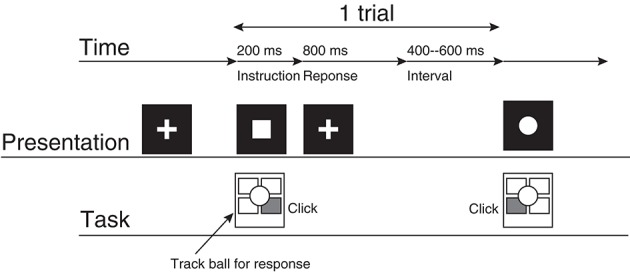
**The experimental procedure**. This represents the participant's task for a case in which the square corresponds to the right click and the circle corresponds to the left click.

The participants performed a two-choice response time (TCRT) task (Figure 1) without feedback about response accuracy (Mars et al., 2008; Kolossa et al., 2012): Two visual stimuli were presented about every 1.5 s, and the participants were required to respond to each stimulus with the previously associated button as quickly as possible. Visual stimuli were presented on an LCD display [VIEWPixx EEG (VPixx Technologies)] with Psychtoolbox-3 and MATLAB R2011b (The MathWorks, Inc.). The participants were seated in front of the display. They touched their left or right hand to the left or right button of a four-button trackball on the desk between the participant and the display.

A single trial consisted of the following procedures. First, the participant gazed at the fixation cross (2.0° × 2.0°) presented at the center of the display for 400–600 ms. Then, the fixation cross vanished, and a circle or a square (2.0° × 2.0°) was presented for 200 ms at the location where the fixation cross had originally been presented. The participant clicked the left or right button as quickly as possible. The participant's response was accepted from 200 ms to 1000 ms after the symbol was presented. If the participant did not respond during this period, then the trial was recorded as a no-response trial. The fixation cross was presented when the symbol vanished.

The symbol-response assignment and instructions were given to the participants before the experiment. For example, participants were instructed “to click the left (right) button when the circle (square) appears.” Before the measurement blocks, the participants underwent a training blocks for the response task consisting of 50 trials; the training blocks was repeated until the participants' response accuracy reached 90%. During the training blocks, the stimulus sequences were generated randomly with a uniform probability distribution.

**Figure 2:**
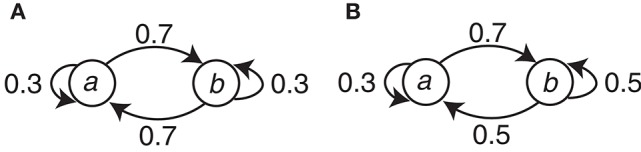
**The Markov chains used to generate the event sequences in the two conditions. (A)** Condition *C1*. **(B)** Condition *C2*.

The symbol stimuli (circle and square) were generated with simple Markov chains. There were two conditions (*C1* and *C2*) with different Markov chains. Let Event *a* and Event *b* correspond to either the circle or the square, respectively, and let *E*_*n*_ be the present event and *E*_*n*−1_ be the preceding event. This assignment is denoted as the event-symbol assignment. Condition *C1* can be represented with the transition probabilities *P*(*E*_*n*_ | *E*_*n*−1_) as *P*(*a* | *a*) = *P*(*b* | *b*) = 0.3 and *P*(*b* | *a*) = *P*(*a* | *b*) = 0.7. For Condition *C2*, *P*(*a* | *a*) = 0.3, *P*(*b* | *a*) = 0.7, and *P*(*a* | *b*) = *P*(*b* | *b*) = 0.5. The Markov chains for the two conditions are summarized in Figure 2.

A single block consisted of 300 trials, and the participants executed a total of four blocks. Two blocks were of Condition *C1*, and two were of Condition *C2*. The event sequence was the same for the two blocks that had the same condition. The participants took a break of at least two minutes between blocks. The participants were not told that there were two conditions for the generative model, and according to a question that we asked the participants after the experiment, none noticed that there were two conditions.

The symbol-response and event-symbol assignments and the block orders were decided randomly as follows. The symbol-response assignment was different for each participant. The event-symbol assignment was chosen randomly during the blocks. The order of the blocks with the two conditions was random.

#### 2.1.3. EEG acquisition

The EEG signals were recorded using a BioSemi ActiveTwo system. The EEG recording was performed at a sampling rate of 512 Hz with a 64-electrode cap, referenced to the common mode sense (CMS) active electrode. The 64 active electrodes were positioned to cover the whole head according to the extended International 10/10 system. The signals in the electrodes placed on the left and right earlobes, on the right side of the right eye (on the temple), and at the left, upper, and lower sides of the left eye were also measured. For preprocessing, the signals were re-referenced with the averaged potential of both earlobes. Moreover, a Butterworth bandpass filter (passband: 0.3–30 Hz, order: 4) was applied to the signals. Epochs were corrected using the −100 to 0 ms period as the baseline. The epochs in which the vertical electroculograms were more than ±80 μV were removed.

### 2.2. Analysis of behavior and EEG

In our analysis, the symbols *aa*, *ab*, *ba*, and *bb* represent the data for the present stimulus after the preceding stimulus. For example, *ab* represents the data for Event *b* after Event *a*.

For the behavioral data, the clicked buttons and the response times of the participants' responses for all trials were acquired. The trials in which the participants responded with the wrong button were removed from the analysis of the response time. We tested the averaged behavioral data for each participant with a two-way repeated-measures analysis of variance (ANOVA) (Cohen et al., 2003; Rac-Lubashevsky and Kessler, 2016) with the factors *Present* (the present stimulus with two levels: Event *a* and Event *b*) and *Preceding* (the preceding stimulus with two levels: Same and Different as the present stimulus). The combinations of the two factors resulted in the sequences *aa* for (Event *a*, Same), *ba* for (Event *a*, Difference), *bb* for (Event *b*, Same), and *ab* for (Event *b*, Difference).

For conventional analysis of ERPs, the trials in which the participants responded with the wrong button were removed from the analysis. We tested the averaged EEG potential at each channel and latency for each participant with an ANOVA in which the factors were the same as those for the behavioral data.

### 2.3. Model-based analysis

For finding the time periods and electrodes in the EEG signals that are well explained by trial-by-trial surprises, we used a model-based analysis of regression with a generalized linear model (GLM) (Bolker et al., 2009). Trial-by-trial surprises were estimated based on the preceding series of the stimulus. The two types of surprise were compared in their effects on the EEG potentials: surprise generated by stationary-state models and by state transition models. A model-based analysis evaluates the relation between two variables with an indicator representing how much one variable accurately explains and/or predicts the other. In this analysis, the variable used to explain the other variable is called the explanatory variable, and the variable to be explained is called the response variable (Haykin, 2005; Dobson and Barnett, 2011).

**Figure 3:**
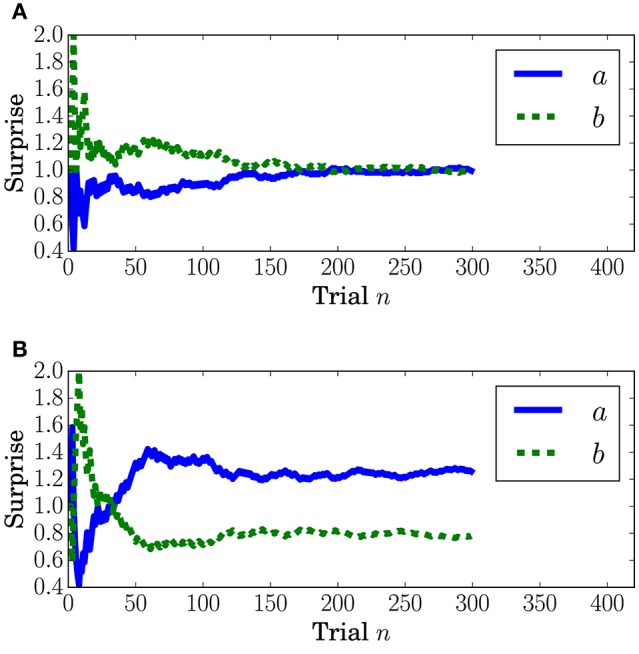
**Predictive stationary surprise *I*_S_ at the *n*th trial**. **(A)** Condition *C1*. **(B)** Condition *C2*.

**Figure 4:**
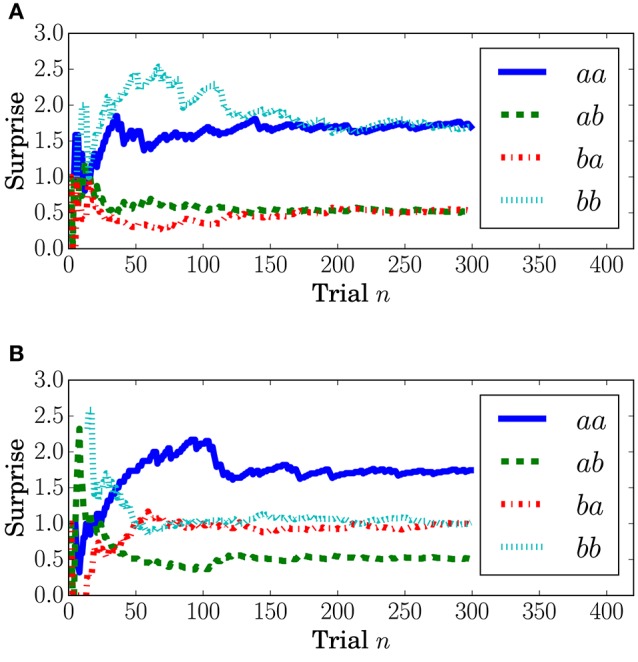
**Transition stationary surprise *I*_T_ at the *n*th trial**. **(A)** Condition *C1*. **(B)** Condition *C2*.

Surprise concerning the present event (called predictive surprise in Kolossa et al., 2012, 2015) was used as the explanatory variable. Predictive stationary surprise is defined as the logarithm of the overall probability given the preceding series of events. Figure 3 shows the trial-by-trial change in predictive stationary surprise for each condition. Additionally, predictive transition surprise is defined as the logarithm of the probability transitioning from the preceding event to the present event given the preceding series of events. Figure 4 shows the trial-by-trial change in predictive transition surprise for each condition. The detailed definitions of surprises are described in Section A.1.

The EEG potentials of each trial were used as the samples of the response variable for the model-based approach. Before the potentials were extracted, the EEG signals over the two blocks of the same condition in each trial were averaged. If either sample in the two blocks was missing because of a wrong task response, no response, or artifact rejection, those trials were removed from the analysis (Kolossa et al., 2012). The trial-by-trial ERPs were extracted by averaging the preprocessed EEG signals over a temporal window of ±50 ms around every 20 ms from 0 to 700 ms from the onset of the stimulus.

The samples of Conditions *C1* and *C2* were merged into a sample set. The merging reduced specific effects of the generative models, such as alternation expectation (Squires et al., 1977; Mars et al., 2008; Kolossa et al., 2012). The number of the samples for each channel and latency was 5578 (the participants' mean = 464.83; and *SD* = 44.56).

**Figure 5:**
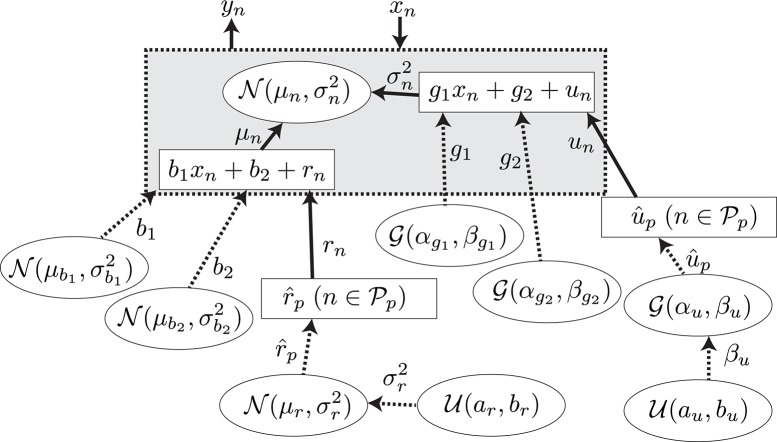
**The graphical model for the model-based analysis**. The variables, the values of which are determined stochastically, are represented by the dashed lines. The deterministic variables are represented by the solid lines. The distributions surrounded by circles represent the non-informative prior probability distributions.

A GLM (Bolker et al., 2009) was adopted for the regression of the explanatory and response variables. The model is summarized in Figure 5. The *N* observed samples of the set of explanatory and response variables (*N* = 5578) are represented as ${\left\{x_{n},y_{n}\right\}}_{n=1}^{N}$, which corresponds to predictive surprise and the EEG potential at a channel and time period for the *n*th sample. The EEG potential is modeled by the linear model of estimated surprise with consideration of individual differences formulated as μ_*n*_ = *b*_1_*x*_*n*_ + *b*_2_ + *r*_*n*_ and an additive Gaussian noise, $N\left(0,\sigma_{n}^{2}\right)$, with the variance formulated as $\sigma_{n}^{2}=g_{1}x_{n}+g_{2}+u_{n}$. The unknown parameters were found with the maximum log-likelihood method (Gelman et al., 2013). Details of the model and the fitting procedure are given in Section A.2.

The fitting accuracy of the regression model was evaluated using the log-Bayes factor of the estimated model. The model $M_{\text{S}}$ was estimated with predictive stationary surprise, and $M_{\text{T}}$ was estimated with predictive transition surprise. Moreover, a common reference model $M_{\text{NULL}}$ was also estimated with a set in which all samples for the response variables were 1 (Neyman and Pearson, 1933; Kolossa et al., 2015). The log-likelihood of $M_{M}$ denoted by log *L*_*M*_ for *M* is S, T, or NULL. As an indicator for the fitting accuracy, the log-Bayes factor *B*_*M*_ with the common reference model (Kass and Raftery, 1995; Kolossa et al., 2015) was adopted:

(1) $$\begin{array}{l} B_{M}=\log L_{M}-\log L_{\text{NULL}}, \end{array}$$

for *M* is S or T.

The log-Bayes factor was evaluated with a likelihood-ratio test (Neyman and Pearson, 1933) that evaluates how more accurately the model fits than the common reference model. A parametric bootstrap method (Davison and Hinkley, 1997) (the number of sampling = 1000) was used for the test.

## 3. Results

### 3.1. Behavioral data

**Figure 6:**
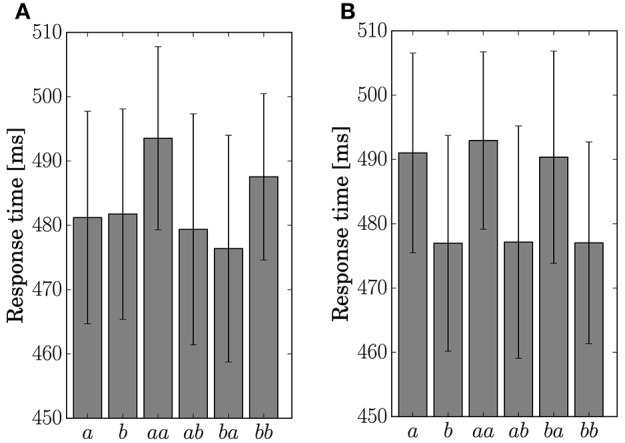
**Response time for the single stimulus or sequence**. The error bars show the standard error. **(A)** Condition *C1*. **(B)** Condition *C2*.

The response time for the button-clicking task was defined as the duration between the display of the stimulus and the clicking of the button. Mean values are displayed in Figure 6. The ANOVA showed a main effect of the factor *Present* in Condition *C2* [*F*_(1, 11)_ = 15.8042, *p* = 0.0022].

**Figure 7:**
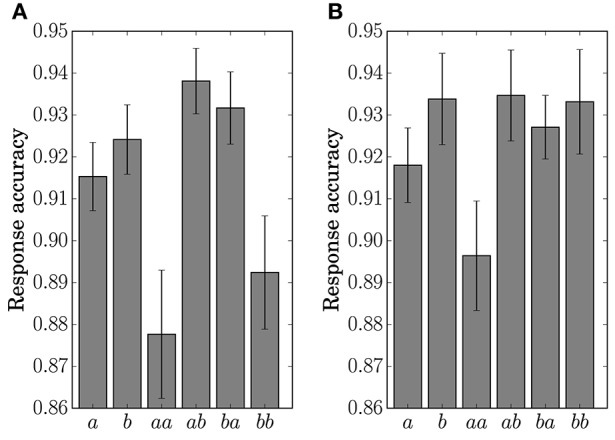
**Response accuracy for the single stimulus or sequence**. The error bars show the standard error. **(A)** Condition *C1*. **(B)** Condition *C2*.

The response accuracy for the button-clicking task was defined as whether or not the participant clicked the assigned button correctly. Mean values are displayed in Figure 7. The ANOVA showed a main effect of the factor *Preceding* in Condition *C1* [*F*_(1, 11)_ = 16.5023, *p* = 0.0019]. In Condition *C2*, main effects were found for the factors *Present* [*F*_(1, 11)_ = 10.1730, *p* = 0.0086] and *Preceding* [*F*_(1, 11)_ = 6.5105, *p* = 0.0269]. An interaction of the two factors [*F*_(1, 11)_ = 6.2011, *p* = 0.03] was also found in Condition *C2*. Simple effects for the interaction were found for *Present* at the level Same [*F*_(1, 11)_ = 14.8977, *p* = 0.0027] and for *Preceding* at the level Event *a* [*F*_(1, 11)_ = 14.9957, *p* = 0.0026].

The results of the statistical analysis suggest that the behavior (response time and accuracy) was affected by state transitions. The main effect of *Preceding* on response accuracy in Condition *C1* reflects the high transition probability for Sequences *ab* and *ba* in the generative model. The difference in the stationary probability between Events *a* and *b* in Condition *C2* can explain the main effects by *Present* for the response time and accuracy. In the response accuracy, the simple effect by *Present* at the level Same corresponds to the difference in the transition probabilities between Sequences *aa* (0.3) and *bb* (0.5) in Condition *C2*. The simple effect by *Preceding* at the level Event *a* corresponds to the difference between Sequences *aa* (0.3) and *ba* (0.5). The differences in the transition probabilities between Sequences *ab* (0.7) and *bb* (0.5), and Sequences *ab* (0.7) and *ba* (0.5) in the generative model of Condition *C2*, however, did not appear in behavior.

### 3.2. Event-related potentials

**Figure 8:**
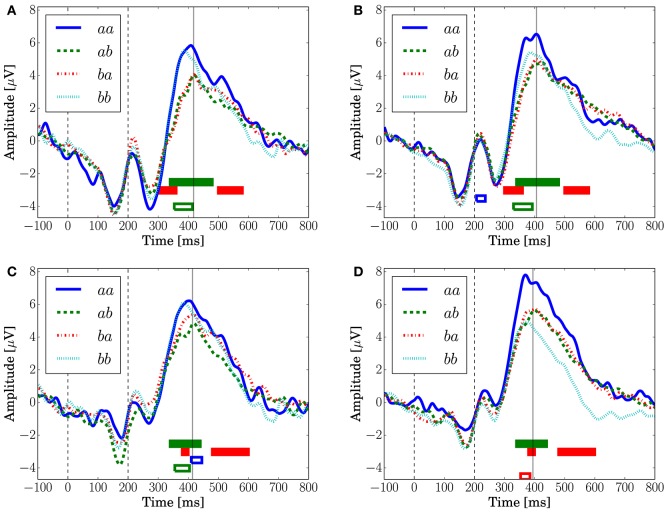
**The grand-averaged EEG potentials observed at the channels FCz and CPz for each sequence with two successive stimuli**. The vertical dashed lines located at 0 and 200 ms represent the onset and offset of the symbol stimulus. The vertical solid lines represents the latency when the maximum amplitude was observed. The time periods in which the main effects are found in the ANOVA at each channel are shown by the red (*Present*), green (*Preceding*), and blue (interaction) unfilled bars (*p* < 0.05). The time periods in which the main effects are found in the log-Bayes factors at each channel are shown by red (the stationary-state model $M_{\text{S}}$) and green (the state transition model $M_{\text{T}}$) filled bars (*p* < 0.05). **(A)** FCz (Condition *C1*). **(B)** FCz (Condition *C2*). **(C)** CPz (Condition *C1*). **(D)** CPz (Condition *C2*).

Figure 8 depicts the grand-averaged ERP waveforms. The ANOVA showed an effect of *Preceding* in FCz (Conditions *C1* and *C2*) and CPz (Condition *C1*) at a latency around 340–400 ms. An effect of *Present* was found in CPz, Condition *C2* at a latency around 370–400 ms. In Figure 8A, the peak amplitude for the sequences that have a transition probability of 0.3 (*aa* and *bb*) is higher than that for those that have a transition probability of 0.7 (*ab* and *ba*). Sequence *aa* in Condition *C2*, which has a transition probability of 0.3, leads to the highest peak amplitude, as shown in Figures 8B,D.

The peaks of P300 are at around 400 ms, which are 100 ms later than the peak latencies reported by Kolossa et al. (2012), who employed a color discrimination task. This difference could be caused by the difference in the stimulus features that an observer should detect for the TCRT tasks (Smid et al., 1999). Mars et al. (2008), who employed a shape discrimination task, reported a similar peak latency as in this analysis, around 400 ms.

### 3.3. Model-based analysis

**Figure 9:**
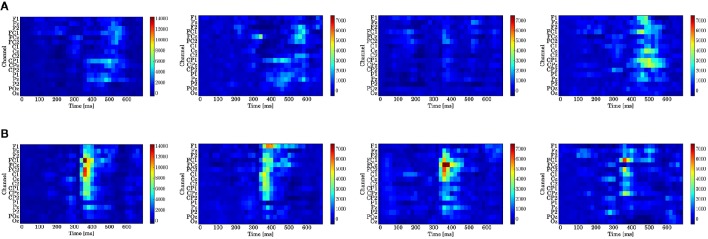
**Log-Bayes factors *B*_*M*_ for each stage of the trials (left to right: whole trials (1st–300th) and early (1st–100th), middle (101st–200th), and last (201st–300th) stages)**. The factor decreases as the color turns from red to blue (see the color bar beside each figure). **(A)** Model based on predictive stationary surprise $M_{\text{S}}$. **(B)** Model based on predictive transition surprise $M_{\text{T}}$.

Figure 9 displays the log-Bayes factors. We found high log-Bayes factors (shown in red), which indicate high fitting accuracy, in some channels and latencies.

For the stationary-state model $M_{\text{S}}$, the likelihood-ratio test showed that the models that reached a log-Bayes factor ≥ 1487 fitted significantly more accurately than the common reference model (*p* < 0.05). The latencies at the channels FCz and CPz in which the statistically significant differences were found are shown as the red bars with the ERP waveforms in Figure 8. At FCz, effects are found within 300–360 and 500–580 ms. At CPz, effects are found within 380–400 and 480–600 ms.

For the state transition model $M_{\text{T}}$, the likelihood-ratio test showed that the models that reached a log-Bayes factor ≥ 2241 were statistically significant (*p* < 0.05). The latencies at the channels FCz and CPz in which the statistically significant differences were found are shown as the green bars with the ERP waveforms in Figure 8. At FCz, an effect is found within 340–480 ms. At CPz, an effect is found within 340–440 ms.

**Figure 10:**
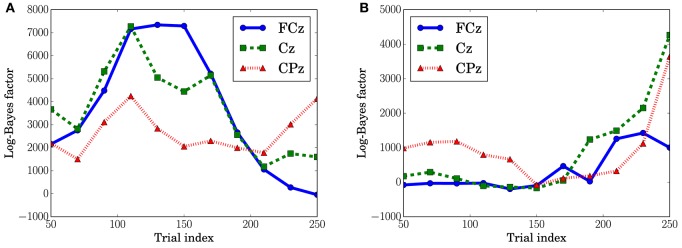
**Log-Bayes factors *B*_*M*_ at the channels, FPz and CPz**. The factors were derived using the ±50th trials from the index of the *x*-axis (e.g., for the trial index 150, the 100th to 200th trials were used). **(A)** Predictive transition surprise, *t* = 360 ms. **(B)** Predictive stationary surprise, *t* = 480 ms.

In Figure 10A, which shows the change in the log-Bayes factors according to the stage of the trials, an increase at the middle stage and a decrease at the last stage in the log-Bayes factors for $M_{\text{T}}$ (*t* = 360 ms) were observed at FCz and Cz. Figure 10B shows that the log-Bayes factor for $M_{\text{S}}$ at Cz and CPz increases as the trials accumulate.

## 4. Discussion

This study investigated the effects of predictive stationary surprise and predictive transition surprise on EEG potentials under the assumption that the internal model is formed with state transitions and predictive surprise is based not only on a stationary-state model but also on a state transition model. For this, we applied Markov chains to generate event sequences in order to isolate the effects of stationary and transition surprises. The results show that predictive stationary surprise better explains P3b and predictive transition surprise better explains P3a. This suggests two distinct mechanisms in human prediction. The effect of predictive transition surprise on P3a suggests that a mechanism for estimating the generative model exists and that the internal model forms a state transition model. The result also indicates a mechanism for processing a stationary-state model as observed by the variability of P3b. The dependencies on time (the number of observed events) of these effects could reflect the process to form the observer's prediction.

We adopted a simple procedure in which predictive surprise was estimated as the self-information of the present event. The self-information for the event was estimated from the preceding event sequences. This procedure is equivalent to the procedure proposed by Mars et al. (2008). However, the optimization problem for the parameters in the DIF model is very complex, and the optimization needs to use an empirical procedure, which does not have the guarantee of a global optimum. Since we focused on the effects on the brain activity that differs between the stationary-state and state transition probabilities, we adopted a fairly simple model, one that does not have any parameters that need to be optimized. Moreover, the DIF model does not accurately produce surprise associated with the state transition model because it is based on a linear combination of the three factors.

The results show that the behavioral data (response accuracy and response time), ERP waveforms, and log-Bayes factors depend on predictive stationary surprise. The behavioral data results correspond to those of Miller (1998) and Kolossa et al. (2012). In the ERP results, as Polich (2007) suggested, the ERP at 390 ms in the centro-parietal region dependent on the stationary probability can be observed. From the model-based analysis, the high fitting accuracy with a centro-parietal focus within 480–600 ms can be considered to be a result of a variation in the P3b component. This speculation is supported by Kolossa et al. (2015), who suggested that P3b is more strongly associated with predictive stationary surprise than P3a.

The effects of predictive transition surprise can be seen in the present results. The behavioral results suggest that response accuracy and response time depend on the preceding event even if the present event is the same: The difficulty of the response depends on the transition probability distribution. The feature observed in the ERP waveforms (Figure 8), that high transition surprise leads to a high peak, is similar to ERP responses to stationary surprise. We suggest here that the effect of the transition probability on behavior and ERPs has not been revealed clearly. In the model-based analysis, the high fitting accuracy with a central focus within 340–480 ms can be considered to be caused by a variation in P3a because similar features in its area (Kopp and Lange, 2013) and latency (Kolossa et al., 2015) have been reported.

The dependence of the P3a component on predictive transition surprise suggests that the participants estimated state transition models as the generative model. This can be explained by introducing Bayesian surprise. This suggests that the variation in P3a occurs via the updating of the internal model. Because the P3a, and thus the update, can be modeled better by predictive transition surprise than by predictive stationary surprise in this experimental setting, it appears that the internal model is associated more strongly with a model with state transitions than with a stationary model. Namely, the internal model approximates a Markov chain. This speculation is supported by the decrease in the fitting accuracy for P3a in $M_{\text{T}}$ at the last stage (Figure 10A) because the internal model converges by accumulating the event observations and Bayesian surprise is slight in the last stage. This convergence corresponds to the convergence in predictive transition surprise shown in Figure 4.

The effect of predictive stationary surprise shows that human prediction has a mechanism different from that of the generative model. Although the internal model is built based on the state transition model, the P3b components depend on the stationary probability distribution. This result suggests that P3b is not affected by the state transitions and mainly reflects the stationary state. The effect of stationary-state models on P3b has been confirmed by El Karoui et al. (2015) and Bekinschtein et al. (2009) as *the global effect*. The increase of the fitting accuracy for P3b at the last stage is consistent with a feature of the global effect that is related to the accumulation of an event on longer time scales (d'Acremont et al., 2013; El Karoui et al., 2015).

Although Kolossa et al. (2015) showed that P3b is distributed in the centro-parietal region, the effect of the stationary-state model is observed also in fronto-central region. This effect could be caused by a P300 latency shift that novelty detection (Courchesne et al., 1975; Knight, 1984) and attention (Kahneman, 1973) affect. This hypothesis is supported by the effect observed in both time periods of the ascending and descending flanks of P300 as shown in Figures 8A,B.

The electrophysiological effects of the two generative models we tested in this study, the stationary-state and state transition models, support theoretical frameworks regarding ERPs, such as the context-updating model, predictive coding, and the Bayesian brain hypothesis. Moreover, the effects suggest the following hypotheses about what kind of models the brain adopts as the internal model. (1) If the external generative model has state transitions, then the internal model can represent the state transitions. (2) If the external generative model does not change over time, then updating of internal model ceases at a certain point. (3) P3b considered to be affected by prediction errors (Spratling, 2010; Kolossa et al., 2012) does not directly reflect the errors between a present event and a prediction generated by the internal model—the P3b variability is caused by the prediction errors for a stationary-state model translated from the internal model or is led by a different process from the generating of the internal model.

As pointed out in Mars et al. (2008) and Kolossa et al. (2012), the TCRT task requires motor responses. Therefore, it is still an open problem whether the cause of the variation in the ERP components is surprise conveyed by the stimulus or surprise associated with the motor responses.

We conclude that our approach using Markov chains provides observation of the different effects on ERPs produced by surprises on the stationary-state and state transition models. The differences in the effects suggest that an internal model in the brain can form a probability model with state transitions. The effects of a stationary-state model suggest the existence of a different brain mechanism from that for forming the internal model. Moreover, a change in these effects by the accumulation of events was observed. This shows the part of neural responses that reflects a brain mechanism by which humans gain predictions from their experiences.

## Author contributions

HH, TM, and SN designed the work. HH and TM collected data. HH analyzed data. HH drafted the manuscript. TM and SN revised the manuscript.

### Conflict of interest statement

The authors declare that the research was conducted in the absence of any commercial or financial relationships that could be construed as a potential conflict of interest.

## A. Details of the model-based analysis

### A.1. Predictive surprise

Given an event sequence composed of *n* stimuli *E*_0_, *E*_1_, …, *E*_*n*−1_ where $E_{n^{\prime }}\in \left\{a,b\right\}$ for *n*′ = 0, …, *n* − 1, surprise concerning the *n*th trial that is based on the stationary or transition probability is defined as follows.

The stationary probability that *E*_*n*_ is *X* is denoted by *P*_*n*_(*X*), defined by

(A1) $$\begin{array}{l} P_{n}(X)=P_{n}(X|{\left\{E_{n^{\prime }}\right\}}_{n^{\prime }=0}^{n-1})\\ \;=\frac{\left|\left\{E_{n^{\prime }}|E_{n^{\prime }}=X,n^{\prime }=0,\ldots ,n-1\right\}\right|}{n}, \end{array}$$

where *X* ∈ {*a, b*}. Then, predictive stationary surprise *P*_*n*_(*X*) is defined by

(A2) $$I_{n}^{\left(\text{S}\right)}=\;\log_{2}P_{n}(E_{n}).$$

The transition probability that *E*_*n*_ is *X* is denoted by *P*_*n*_(*X* | *E*_*n*−1_), defined as

(A3) $$\begin{array}{l} \;P_{n}(X|E_{n-1})\\ =P_{n}(X|E_{n-1},{\left\{E_{n^{\prime }}\right\}}_{n^{\prime }=0}^{n^{\prime }-2})\\ =\frac{\left|\left\{E_{n^{\prime }}|E_{n^{\prime }}=X,E_{n^{\prime }-1}=E_{n-1},n^{\prime }=0,\ldots ,n-1\right\}\right|}{\left|\left\{E_{n^{\prime }}|E_{n^{\prime }}=X,n^{\prime }=0,\ldots ,n-1\right\}\right|}. \end{array}$$

Then predictive transition surprise *P*_*n*_(*X* | *E*_*n*−1_) is defined as

(A4) $$I_{n}^{\left(\text{T}\right)}=\log_{2}P_{n}(E_{n}|E_{n-1}).$$

### A.2. Regression model

In this section, we describe the regression model summarized in Figure 5. We assume that the samples of the response variable are generated with a Gaussian distribution by

(A5) $$y_{n}\sim N(\mu_{n},\sigma_{n}^{2}),$$

where $N\left(\mu ,\sigma^{2}\right)$ represents a Gaussian distribution with mean μ and variance σ^2^. The parameters of the distribution are assumed to be

(A6) $$\mu_{n}=b_{1}x_{n}+b_{2}+r_{n},$$

and

(A7) $$\sigma_{n}^{2}=g_{1}x_{n}+g_{2}+u_{n},$$

where *b*_1_ and *g*_1_ are the slopes, *b*_2_ and *g*_2_ are the intercepts of the model, and *r*_*n*_ and *u*_*n*_ are the individual differences of the participants. Let $P_{p}$ be the set of the indexes of the samples obtained from the participant *p*, where $P_{i}\cap P_{j}=\emptyset$ (*i, j* = 1, …, *N*_*P*_, *i* ≠ *j*), $P_{1}\cup P_{2}\cup \cdots \cup P_{N_{P}}=\left\{1,\ldots ,N\right\}$, and *N*_*P*_ is the number of participants. Then, *r*_*n*_ and *u*_*n*_ are defined as

(A8) $$r_{n}={\hat{r}}_{p},\;n\in P_{p}$$

and

(A9) $$u_{n}={\hat{u}}_{p},\;n\in P_{p}.$$

Therefore, the unknown parameters for the individual difference in the model are ${\left\{{\hat{r}}_{p},{û}_{p}\right\}}_{p=1}^{N_{P}}$, not ${\left\{r_{n},u_{n}\right\}}_{n=1}^{N}$. Moreover, we assume the priors for *b*_1_, *b*_2_, *g*_1_, *g*_2_, ${\left\{{\hat{r}}_{p}\right\}}_{p=1}^{N_{P}}$, and ${\left\{{ŝ}_{p}\right\}}_{p=1}^{N_{P}}$ to be

(A10) $$b_{1}\;\sim \;N(\mu_{b_{1}},\sigma_{b_{1}}^{2}),$$

(A11) $$b_{2}\;\sim \;N(\mu_{b_{2}},\sigma_{b_{2}}^{2}),$$

(A12) $$g_{1}\;\sim \;G(\alpha_{g_{1}},\beta_{g_{1}}),$$

(A13) $$g_{2}\;\sim \;G(\alpha_{g_{2}},\beta_{g_{2}}),$$

(A14) $${\hat{r}}_{p}\sim N(\mu_{r},\sigma_{r}^{2}),\;p=1,\ldots ,N_{P},$$

and

(A15) $${\hat{u}}_{p}\sim G(\alpha_{u},\beta_{u}),\;p=1,\ldots ,N_{P},$$

where G(α,β) is a gamma distribution with shape α and scale β. Furthermore, we define the hyper priors for $\sigma_{r_{p}}^{2}$ and β_*u*_*p*__ as

(A16) $$\sigma_{r}^{2}\sim U(a_{r},b_{r}),$$

and

(A17) $$\sigma_{u}^{2}\sim U(a_{u},b_{u}),$$

where U(a,b) is the uniform distribution over the interval [*a, b*]. The undefined parameters for the model were assumed to be constants. The parameters shown in Table A1 were set to give a non-informative prior distribution for the parameters.

**Table A1:** **The parameters that we consider to be constants in the model (Figure 5) and their values**.

**Parameter**	**Value**	**Parameter**	**Value**
μ_*b*_1__	0	σ_*b*_1__	100
μ_*b*_2__	0	σ_*b*_2__	100
α_*g*_1__	1	β_*g*_1__	100
α_*g*_2__	1	β_*g*_2__	100
μ_*r*_	0	α_*u*_	1
*a*_*r*_	0	*b*_*r*_	100
*a*_*u*_	0.1	*b*_*u*_	100

We found the unknown parameters for the hierarchical model by sampling with the Markov chain Monte Carlo (MCMC) method (Gelman et al., 2013). In particular, we used the Metropolis–Hastings algorithm implemented in PyMC 2.3.6 (Patil et al., 2010) for the sampling. The number of sampling was 100,000 (burn-in: 10,000).
